# Body mass index and basal androstenedione are independent risk factors for miscarriage in polycystic ovary syndrome

**DOI:** 10.1186/s12958-018-0438-7

**Published:** 2018-11-19

**Authors:** Wan Yang, Rui Yang, Mingmei Lin, Yan Yang, Xueling Song, Jiajia Zhang, Shuo Yang, Ying Song, Jia Li, Tianshu Pang, Feng Deng, Hua Zhang, Ying Wang, Rong Li, Jie Jiao

**Affiliations:** 1 Center for Reproductive Medicine, Department of Obstetrics and Gynecology, Peking University Third Hospital, 49 N Garden Rd, Haidian District, Beijing, 100191 China; 2 Research Center of Clinical Epidemiology, Peking University Third Hospital, Beijing, China

**Keywords:** Gonadotropin-releasing hormone antagonist, Polycystic ovary syndrome, Hyperandrogenism, Body mass index, In vitro fertilization

## Abstract

**Background:**

There is limited literature investigating the effects of body mass index (BMI) and androgen level on in vitro fertilization (IVF) outcomes with a gonadotropin-releasing hormone (GnRH)-antagonist protocol in polycystic ovary syndrome (PCOS). Androgen-related variation in the effect of body mass index (BMI) on IVF outcomes remains unknown.

**Methods:**

In this retrospective study, 583 infertile women with PCOS who underwent IVF using the conventional GnRH-antagonist protocol were included. Patients were divided into four groups according to BMI and androgen level: overweight- hyperandrogenism(HA) group, *n* = 96, overweight-non-HA group, *n* = 117, non-overweight-HA group, *n* = 152, and non-overweight-non-HA group, *n* = 218.

**Results:**

A significantly higher number of oocytes were retrieved, and the total Gn consumption as well Gn consumption per day was significantly lower, in the non-overweight groups than in the overweight groups. The number of available embryos was significantly higher in the HA groups than in the non-HA groups. Clinical pregnancy rate was of no significant difference among four groups. Live-birth rates in the overweight groups were significantly lower than those in non-overweight-non-HA group (23.9, 28.4% vs. 42.5%, *P*<0.05). The miscarriage rate in overweight-HA group was significantly higher than that in non-overweight-non-HA group (45.2% vs. 14.5%, *P*<0.05). Multivariate logistic regression analysis revealed that BMI and basal androstenedione (AND) both acted as significantly influent factors on miscarriage rate. The area under the curve (AUC) in receiver operating characteristic (ROC) analysis for BMI and basal AND on miscarriage rate were 0.607 (*P* = 0.029) and 0.657 (*P* = 0.001), respectively, and the cut-off values of BMI and basal AND were 25.335 kg/m^2^ and 10.95 nmol/L, respectively.

**Conclusions:**

In IVF cycles with GnRH-antagonist protocol, economic benefits were seen in non-overweight patients with PCOS, with less Gn cost and more retrieved oocytes. BMI and basal AND were both significantly influential factors with moderate predictive ability on the miscarriage rate. The predictive value of basal AND on miscarriage was slightly stronger than BMI.

## Introduction

For infertile women with polycystic ovary syndrome (PCOS) who fail lifestyle intervention and ovulation induction therapy or who have additional infertility factors, in vitro fertilization (IVF) can be used. Moderate evidence suggests that a gonadotropin (Gn)-releasing hormone (GnRH)-antagonist protocol can significantly reduce the incidence of ovarian hyperstimulation syndrome (OHSS) [1–3], and the use of a GnRH-antagonist protocol is gradually being adopted by clinicians. Phenotypic expressions of PCOS include oligo-ovulation/anovulation, hyperandrogenism (HA), polycystic ovaries, overweight/obesity, and insulin resistance/metabolic syndrome [4]. There is limited literature investigating the clinical phenotype of patients with PCOS who can benefit the most from a GnRH-antagonist protocol during IVF.

In China, 34.63% of patients with PCOS have a body mass index (BMI) above 23 kg/m^2^ [5]. There are different opinions about the role of BMI in IVF outcomes. In 2016, Provost et al. [6] analyzed a total of 239,127 fresh IVF cycles and demonstrated a progressive and statistically significant worsening of outcomes in groups with higher BMIs, including cycles performed specifically for PCOS or male-factor infertility. Bailey et al. [7] also indicated that obese women with PCOS had lower odds of clinical pregnancy and live birth than lean women with PCOS, while there was a trend toward decreased OHSS incidence with increasing BMI among women with PCOS. However, Dechaud et al. [8] showed that obese patients required a higher recombinant follicle-stimulating hormone (r-FSH) dose, but it did not have any adverse impact on IVF, including the cancellation rate, implantation rate, and pregnancy rate. Metwally et al. [9] found that obesity adversely affected embryo quality in young women undergoing IVF/intracytoplasmic sperm injection, while the oocyte quality was not affected.

The prevalence of biochemical HA in patients with PCOS is 78.2% [10]. Studies on the role of HA in IVF outcomes are limited. A recent study reported that HA in women with PCOS did not compromise IVF results, in contrast, facilitated the retrieval of a significantly higher number of oocytes [11]. In our recent study, HA also had a positive effect on the number of retrieved oocytes, where it is associated with miscarriage rate as well [12]. Conversely, another study inferred that the combination of HA and chronic anovulation was associated with a negative impact on the clinical pregnancy rate in patients with PCOS [13]. Endocrine disturbances are complicated in patients with PCOS. HA and obesity interact with each other and promote the progression of PCOS. However, few studies to date have investigated BMI and androgen level together. Therefore, the present study was designed to evaluate whether the effect of BMI on IVF outcomes vary with the level of androgen in PCOS with a GnRH-antagonist protocol.

## Materials and methods

Between January 2013 and December 2015, a total of 583 infertile patients with PCOS treated with the conventional GnRH-antagonist protocol at the Center for Reproductive Medicine of Peking University Third Hospital were screened and enrolled in the study. Patients with PCOS between 20 and 35 years of age who were undergoing their first IVF cycle were included. Exclusion criteria included endometriosis, previous ovarian surgical history, hydrosalpinx, severe oligoasthenospermia or azoospermia, and systemic diseases such as diabetes mellitus and hypo- or hyperthyroidism. This retrospective cohort study was approved by the Institutional Review Board of the Department of Obstetrics and Gynecology, Peking University Third Hospital of Beijing, China.

In all cases, PCOS was diagnosed according to the Rotterdam 2003 criteria [14]. BMI was calculated by the following formula: BMI = weight/height^2^ (kg/m^2^). HA was diagnosed by a testosterone level ≥ 2.2 nmol/L or AND level ≥ 12 nmol/L. Testosterone and AND levels were obtained from basal sex hormone assessments. This cohort study included four groups of patients with PCOS: BMI ≥ 25 kg/m^2^ with HA (overweight-HA group, *n* = 96), BMI ≥ 25 kg/m^2^ with normal androgen (overweight-non-HA group, *n* = 117), BMI < 25 kg/m^2^ with HA (non-overweight-HA group, *n* = 152), and BMI < 25 kg/m^2^ with normal androgen (non-overweight-non-HA group, *n* = 218).

All patients were stimulated with a combination of a GnRH antagonist (cetrorelix; SeronoMunich, Germany) and an r-FSH drug (Gonal-f; Merck Serono, Munich, Germany) to develop multiple follicles. Controlled ovarian hyperstimulation was started from 2nd day of the menstrual period when no follicle > 10 mm in diameter was detected by ultrasound, and serum estradiol levels were < 50 pg/mL. The Gn dose was modified in accordance with the ovarian response. Highly purified human menopausal gonadotropin (menogon; Ferring, Kiel, Germany) was occasionally added. A GnRH antagonist was injected subcutaneously at a daily dose of 0.25 mg when follicles with a mean diameter ≥ 14 mm were detected. The criterion for administration of recombinant human chorionic gonadotropin (hCG) (250 μg) was the observation of at least two follicles with a mean diameter ≥ 18 mm. Thirty-six hours after the trigger, oocyte retrieval was performed with ultrasound guidance, using a 16-G double-lumen aspiration needle. Conventional fertilization and embryo culture were performed. In cases where the number of retrieved oocytes was ≥20 or estradiol levels were above 15,000 pmol/L on the day of hCG administration, all available embryos were cryopreserved by the vitrification method for future transfer to prevent OHSS. Two embryos were transferred three days after oocyte retrieval. Supportive therapy with progesterone was administered vaginally (90 mg daily), starting on the day of oocyte collection, and was continued until 10 weeks of gestation.

Primary outcome measures were rate of clinical pregnancy, miscarriage rate, and rate of live birth. Secondary outcome measures were Gn dosage, number of oocytes collected, and number of available embryos.

Data were analyzed with SPSS 20.0 (Chicago, IL). The chi-square test was used for categorical variables, and analysis of variance was used for continuous variables. Logistic regression analysis was performed using these binary variables in a forward stepwise method. The optimal cut-off values were calculated by receiver operating characteristic (ROC) analysis using the Youden index. A *P* value of < 0.05 was considered statistically significant for all analyses.

## Results

Baseline characteristics of patients are shown in Table 1. There were no significant differences in age, basal FSH, and antral follicle count between the groups. The duration of infertility was significantly higher in the overweight groups than in the non-overweight groups. The basal luteinizing hormone (LH) levels were significantly higher in the HA groups than in the non-HA groups.

**Table 1:** Baseline characteristics of patients

Parameter	Non-overweight		Overweight		*P* value
	Non-HA (*n* = 218)	HA (*n* = 152)	Non-HA (*n* = 117)	HA (*n* = 96)	
Age (y)	29.2 ± 3.6	29.0 ± 3.4	29.8 ± 3.2	29.1 ± 3.2	*NS*
BMI (kg/m^2^)	21.5 ± 2.1^a^	21.6 ± 2.2 ^a^	28.5 ± 2.3^b^	28.7 ± 3.1 ^b^	*<0.001*
Infertile duration (y)	3.7 ± 2.5 ^a^	3.7 ± 2.1 ^a^	4.6 ± 2.7 ^b^	4.4 ± 2.8 ^b^	*<0.001*
Basal FSH (IU/L)	6.1 ± 1.5	6.3 ± 1.8	5.9 ± 1.8	6.1 ± 1.7	*NS*
Basal LH (IU/L)	5.7 ± 3.8^a^	9.7 ± 5.9 ^b^	5.5 ± 3.8^a^	8.9 ± 4.6^b^	*<0.001*
Basal T(ng/ml)	0.8 ± 0.3^a^	1.4 ± 1.0 ^b^	0.9 ± 0.3^a^	2.0 ± 3.0^c^	*<0.001*
Basal AND (nmol/L)	8.0 ± 2.5^a^	17.3 ± 6.0 ^b^	8.6 ± 2.3^a^	19.2 ± 7.3^c^	*<0.001*
Total AFC (n)	19.0 ± 6	19.8 ± 5	19.1 ± 6	20.8 ± 7	*NS*

Data are presented as mean ± standard deviation unless otherwise specified

*NS* not significant; *HA* hyperandrogenism; *AFC* antral follicle count; *BMI* body mass index; *FSH* follicle-stimulating hormone; *LH* luteinizing hormone; *T* testosterone; *AND* androstenedione

^a^ Significantly different from ^b^ or ^c^ groups

Characteristics of ovarian responses among the four patient groups are shown in Table 2. The number of oocytes retrieved and the estradiol levels on the day of hCG administration were significantly higher in the non-overweight groups than in the overweight groups, although the total Gn consumption, as well as Gn consumption per day, was significantly lower in the non-overweight groups. Despite the effect of BMI, Gn consumption per kilogram was significantly lower in the HA groups. As with the number of oocytes retrieved, the cancellation rates of embryo transfer to prevent OHSS were significantly higher in the non-overweight groups than in the overweight groups. Unlike the basal LH levels that were related to basal androgen levels, LH levels on the day of hCG administration were significantly lower in the non-overweight groups than in the overweight groups. The number of available embryos was higher in non-overweight-HA group than in overweight-non-HA group. The level of progesterone (P4) and the endometrial thickness on the day of hCG administration were not different among the four groups. Similarly, the fertilization rate and the cleavage rate were comparable among the four groups.

**Table 2:** Characteristics of ovarian responses

Parameter	Non-overweight		Overweight		*P* value
	Non-HA (*n* = 218)	HA (*n* = 152)	Non-HA (*n* = 117)	HA (*n* = 96)	
Gn stimulation days	10.5 ± 2.1^a^	9.8 ± 1.8^b^	11.3 ± 2.6^c^	10.5 ± 2.4^a^	*<0.001*
Total Gn dosage (IU)	1588 ± 659^a^	1420 ± 595^a^	2081 ± 885^b^	1828 ± 750^c^	*<0.001*
Gn dosage /day (IU)	148 ± 40^a^	141 ± 35^a^	180 ± 49^b^	170 ± 40^c^	*<0.001*
Gn consumption/kg (IU)	28.3 ± 12^a^	25.1 ± 10^b^	27.6 ± 11	24.9 ± 10^b^	*<0.05*
Gn consumption/ kg/day	2.6 ± 0.7^a^	2.5 ± 0.6	2.4 ± 0.6	2.3 ± 0.5^b^	*<0.001*
E2 on HCG day (pmol/L)	12,708 ± 7621^a^	13,663 ± 8165 ^a^	9219 ± 6685^b^	11,143 ± 7556	*<0.001*
LH on HCG day (mIU/mL)	2.4 ± 2.2^a^	2.4 ± 2.3^a^	3.0 ± 3.6	3.2 ± 2.6^b^	*<0.001*
P4 on HCG day (nmol/L)	2.4 ± 1.4	2.4 ± 1.4	2.2 ± 1.3	2.5 ± 1.5	*NS*
Endometrial thickness	10.7 ± 1.7	10.4 ± 1.7	10.7 ± 1.8	10.2 ± 1.9	*NS*
Oocyte retrieved (n)	16.7 ± 8.4^a^	18.2 ± 9.9^a^	12.1 ± 6.9^b^	13.8 ± 8.5^b^	*<0.001*
ET cancellation rate^d^	26.6%^a^	35.5%^a^	14.5%^b^	18.8%^b^	*<0.05*
Fertilization rate (%)	78.0 ± 19	76.5 ± 19	76.2 ± 24	75.1 ± 21	*NS*
Cleavage rate (%)	96.7 ± 11	98.9 ± 03	98.6 ± 05	97.6 ± 08	*NS*
Available embryos	7.75 ± 5.6	8.91 ± 6.9^a^	5.34 ± 4.7 ^b^	6.68 ± 6.0	*<0.001*

Data are presented as mean ± standard deviation unless otherwise specified

*NS* not significant; *BMI* body mass index; *HA* hyperandrogenemia; *PCOS* polycystic ovary syndrome; *Gn* gonadotropin; *E2* estradiol; *LH* luteinizing hormone; *P4* progesterone; *hCG* human chorionic gonadotropin; *ET* embryo transfer

^a^ Significantly different from ^b^ or ^c^ groups

^d^The cancellation rate of embryo transfer to prevent ovarian hyperstimulation syndrome

Pregnancy outcomes are presented in Table 3. The miscarriage rate was significantly higher in overweight-HA group than in non-overweight-non-HA group. The live-birth rates were significantly lower in the overweight groups than in non-overweight-non-HA group. There were no ectopic pregnancies in HA groups. There were no differences in the rates of clinical pregnancy, preterm pregnancy, twin pregnancy, or caesarean section among the study groups.

**Table 3:** Pregnancy outcomes

Parameter	Non-overweight		Overweight		*P* value
	Non-HA (*n* = 218)	HA (*n* = 152)	Non-HA (*n* = 117)	HA (*n* = 96)	
Clinical PR	49.7% (76/153)	43.6% (41/94)	40.9% (36/88)	43.7% (31/71)	*NS*
Miscarriage rate	14.5% (11/76)^a^	26.8% (11/41)	30.6% (11/36)	45.2% (14/31)^b^	*<0.05*
Live-birth rate	42.5% (65/153)^a^	31.9% (30/94)	28.4% (25/88)^b^	23.9% (17/71)^b^	*<0.05*
Ectopic PR	3.3% (5/153)	0% (0/94)^a^	5.7% (5/88)^b^	0% (0/71)	*<0.05*
Singleton PR	60.5%(46/76)	56.1%(23/41)	58.3% (21/36)	58.1% (18/31)	*NS*
Twin PR	39.5%(30/76)	43.9%(18/41)	41.7% (15/36)	41.9% (13/31)	*NS*
Preterm rate	22.4%(17/76)	19.5%(8/41)	19.4% (7/36)	12.9% (4/31)	*NS*
Full term rate	63.2%(48/76)	53.7%(22/41)	50.0%(18/36)	41.9% (13/31)	*NS*
CS rate	72.3% (47/65)	70.0% (21/30)	84.0% (21/25)	94.1% (16/17)	*NS*

*HA* hyperandrogenemia; *PCOS* polycystic ovary syndrome; *BMI* body mass index; *NS* not significant; *PR* pregnancy rate; *CS* caesarean section

^a^ Significantly different from ^b^ groups

**Fig. 1:**
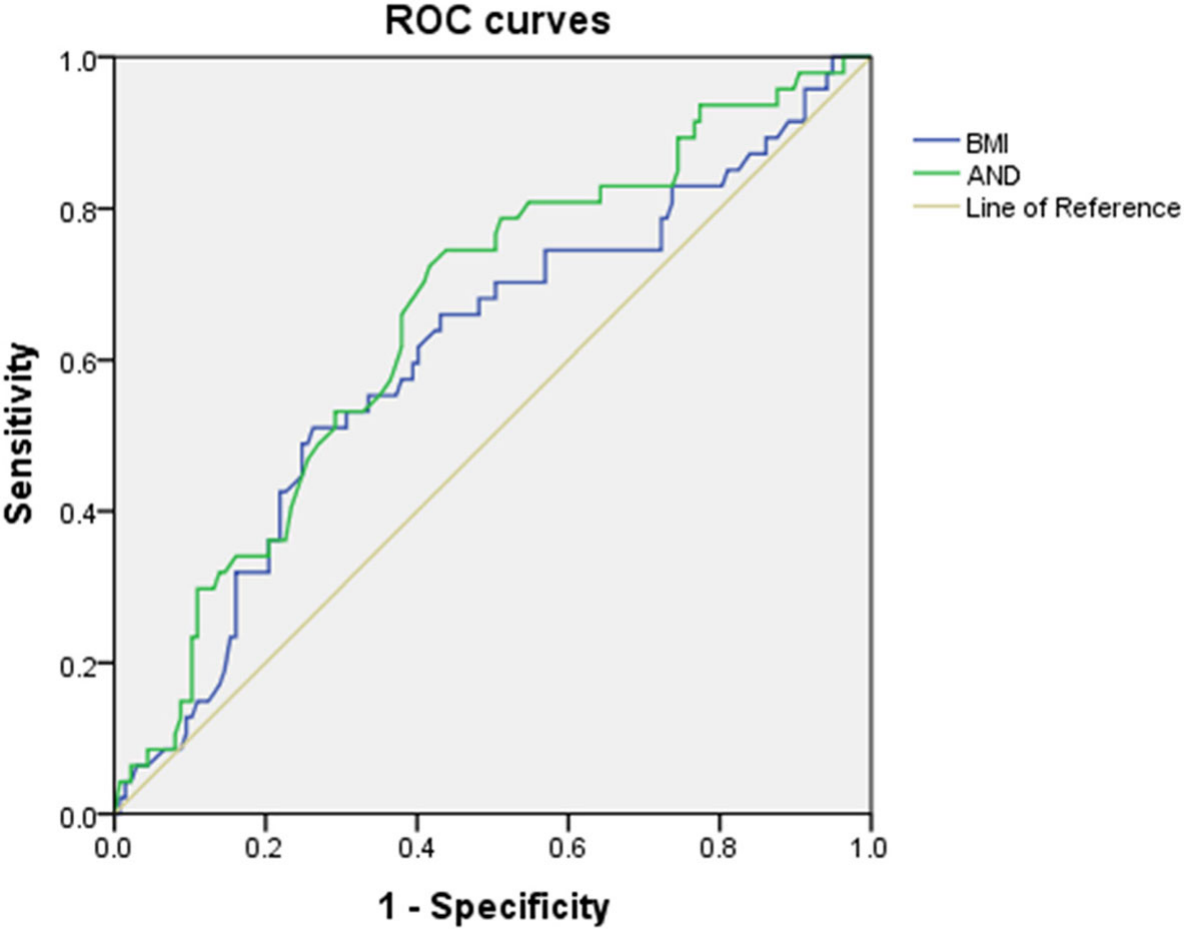
Receiver operating characteristic (ROC) curves analysis of body mass index (BMI) and androstenedione (AND) on prediction of miscarriage rate. The green line and blue lines represent AND and BMI, respectively, and the beige line is the reference

Multivariate logistic regression analyses and ROC analysis were performed to define the predictive factors of miscarriage rate. Parameters that could have adverse effects on miscarriage rate were age, BMI, basal testosterone, basal AND, Gn consumption/kg/day, and number of oocytes retrieved. These parameters, as well as LH levels (both basal LH and LH on the day of hCG administration), were chosen for multivariate logistic regression analyses. After adjusting the effects of other parameters, BMI and basal AND showed independently significant differences in predicting the miscarriage rate (Table 4). A ROC analysis was performed to define the optimal cut-off values of BMI and basal AND (Fig. 1). The areas under the curve (AUCs) for the BMI and basal AND were 0.607 (*P* = 0.029) and 0.657 (*P* = 0.001), respectively (Table 5). These two parameters were demonstrated to have moderate predictive ability on miscarriage, with a sensitivity of 0.511–0.723 and a specificity of 0.584–0.737. Cut-off values were as follows: BMI, 25.335 kg/m^2^; and basal AND, 10.95 nmol/L.

**Table 4:** Multivariate logistic regression analysis for predictive factors of miscarriage rate

Parameter	β	*P* value	Odds ratio	CI(95%)
Age (y)	0.040	0.502	1.040	0.927–1.168
BMI (kg/m^2^)^a^	0.093	0.049	1.097	1.000–1.204
Basal LH (IU/L)	0.030	0.379	1.031	0.963–1.103
Basal T(ng/ml)	0.095	0.684	1.100	0.695–1.742
Basal AND (nmol/L)^a^	0.069	0.034	1.071	1.005–1.141
Gn consumption/kg/day	0.579	0.061	1.785	0.974–3.273
LH on HCG day (mIU/mL)	0.061	0.258	1.063	0.957–1.180
Oocyte retrieved (n)	0.022	0.533	1.023	0.953–1.098
Constant	−7.630	0.001	0.000	

*BMI* body mass index; *LH* luteinizing hormone; *T* testosterone; *AND* androstenedione; *CI* confidence interval; *Gn* gonadotropin; *hCG* human chorionic gonadotropin

^a^Significant difference

**Table 5:** ROC analysis of BMI and AND on prediction of miscarriage rate

Parameter	Cut-off value	AUC	*P* value	Sensitivity	Specificity
BMI (kg/m^2^)	25.335	0.607	0.029	0.511	0.737
Basal AND (nmol/L)	10.95	0.657	0.001	0.723	0.584

*BMI* body mass index; *AND* androstenedione; *ROC* receiver operating characteristic; *AUC* area under the curve

## Discussion

Nearly 70–80% of anovulatory infertility cases are caused by PCOS [15]. As a therapy, IVF can be safely used in infertile women with PCOS, especially with a GnRH-antagonist protocol. There is few studies to date have investigated the parameters of BMI and androgen together with the use of GnRH antagonists. In this study, the aim was to assess Androgen-related variation in the effect of body mass index (BMI) on IVF outcomes.

In this study, the duration of infertility was significantly higher in the overweight groups than in the non-overweight groups, indicating that body weight may contribute to a longer duration of infertility. The number of oocytes retrieved was significantly higher in the non-overweight groups than in the overweight groups, although the total Gn consumption as well as Gn consumption per day was significantly lower. There is an economic benefit of using a GnRH-antagonist protocol in non-overweight patients with PCOS, because of less cost and larger number of retrieved oocytes. Despite the effect of BMI, Gn consumption per kilogram was significantly lower in the HA groups, indicating that when overweight PCOS patients lose weight, patients with HA gain economic benefits. The number of available embryos was higher in non-overweight-HA group than in overweight-non-HA group, indicating that a non-overweight build and over-secretion of androgen may be protective factors for good-quality embryos. The miscarriage rate was significantly higher in overweight-HA group than in non-overweight-non-HA group, indicating that being overweight and having HA may play an important role in higher miscarriage rates. It appeared that being overweight was a risk factor for both low-quality embryos and miscarriage. However, HA was a positively influential factor for good-quality embryos but a risk factor for miscarriage. Meanwhile, in multivariate logistic regression analyses, BMI and basal AND showed independently significant differences on prediction of miscarriage, after considering the effects of other parameters that may cause miscarriage. ROC analysis showed that the optimal cut-off values of BMI and basal AND were 25.335 kg/m^2^ and 10.95 nmol/L, respectively. Although the predictive abilities of both BMI and basal AND on miscarriage were moderate, with a sensitivity of 0.511–0.723 and a specificity of 0.584–0.737, they may still provide predictive values in clinical treatment since the AUCs for BMI and basal AND level were significantly different (*P* = 0.029 and *P* = 0.001, respectively). In addition, the predictive value of basal AND on miscarriage was slightly stronger than that of BMI.

PCOS is closely related to miscarriages. The molecular mechanisms underlying PCOS-associated miscarriages are controversial. Obesity and HA are factors that may contribute to miscarriage [13], which was confirmed by our previous study. By observational analysis of the developmental kinetics and metabolic activity of oocytes, some researchers have observed that oocytes from women who are overweight or obese are smaller than those from women of healthy weight (BMI < 25 kg/m^2^), yet post-fertilization they reach the morula stage faster and, as blastocysts, show reduced glucose consumption and elevated endogenous triglyceride levels [16]. The data indicate that a high BMI in women is associated with distinct phenotypic changes in the embryo, which may reduce the quality of embryos and cause miscarriage. Valckx et al. showed that metabolic alterations in the serum were reflected in the follicular fluid, and that some of these alterations may have affected oocyte quality; however, there were poor BMI-related associations [17]. As for HA-related miscarriages, there are several potential mechanisms. First, the basal LH levels are related to basal androgen levels, as shown in our study. HA contributes to the secretion of excessive amounts of LH that may cause oocyte maturation disturbances and miscarriage. Second, HA may have an adverse effect on ovarian folliculogenesis and granulosa cell function. Follicular atresia is potentiated by androgens in the immature rat, and granulose cell apoptosis in rats is inducible by androgens [18]. Increased expression of AKT1 and AKT2 may be a possible mechanism linking HA to granulosa cell dysfunction in patients with HA PCOS [19]. Finally, testosterone may impact the endometrium. Testosterone was reported to have a dose-dependent negative effect on the proliferation of decidualized endometrial stromal cells [20] and to suppress the expression of HOXA10, which is essential to endometrial receptivity [21]. In patients with PCOS, HA and obesity interact with each other and promote the progression of PCOS.

One limitation of our study is its retrospective design. The effects of BMI and HA are assessed in the cohort study with logistic regression analysis and ROC analysis, showing the optimal cut-off values of BMI and basal AND. Although AUCs in ROC analysis are significantly different, the predictive ability of both parameters on miscarriage is moderate, due to the relatively limited sample size. Prospective studies with a larger sample size are needed in the future, along with studies investigating the effects of hyperinsulinemia.

## Conclusions

In conclusion, being overweight was a risk factor for both low-quality embryos and miscarriage. However, HA was a positively influential factor for good-quality embryos but a risk factor for miscarriage. Multivariate logistic regression analyses show that BMI and basal AND were both significantly influential factors on miscarriage rate, with moderate predictive ability. The predictive value of basal AND on miscarriage was slightly stronger than BMI.
